# Supplementary material for: Regulatory T cells infiltrate the tumor-induced tertiary lymphoïd structures and are associated with poor clinical outcome in NSCLC
Source: Commun Biol. 2022 Dec 24;5:1416. doi: 10.1038/s42003-022-04356-y (PMC9789959; doi:10.1038/s42003-022-04356-y)
Supplement: Supplementary file 2 — Supplementary Information [file 42003_2022_4356_MOESM2_ESM.pdf]

Supplementary Figure 1

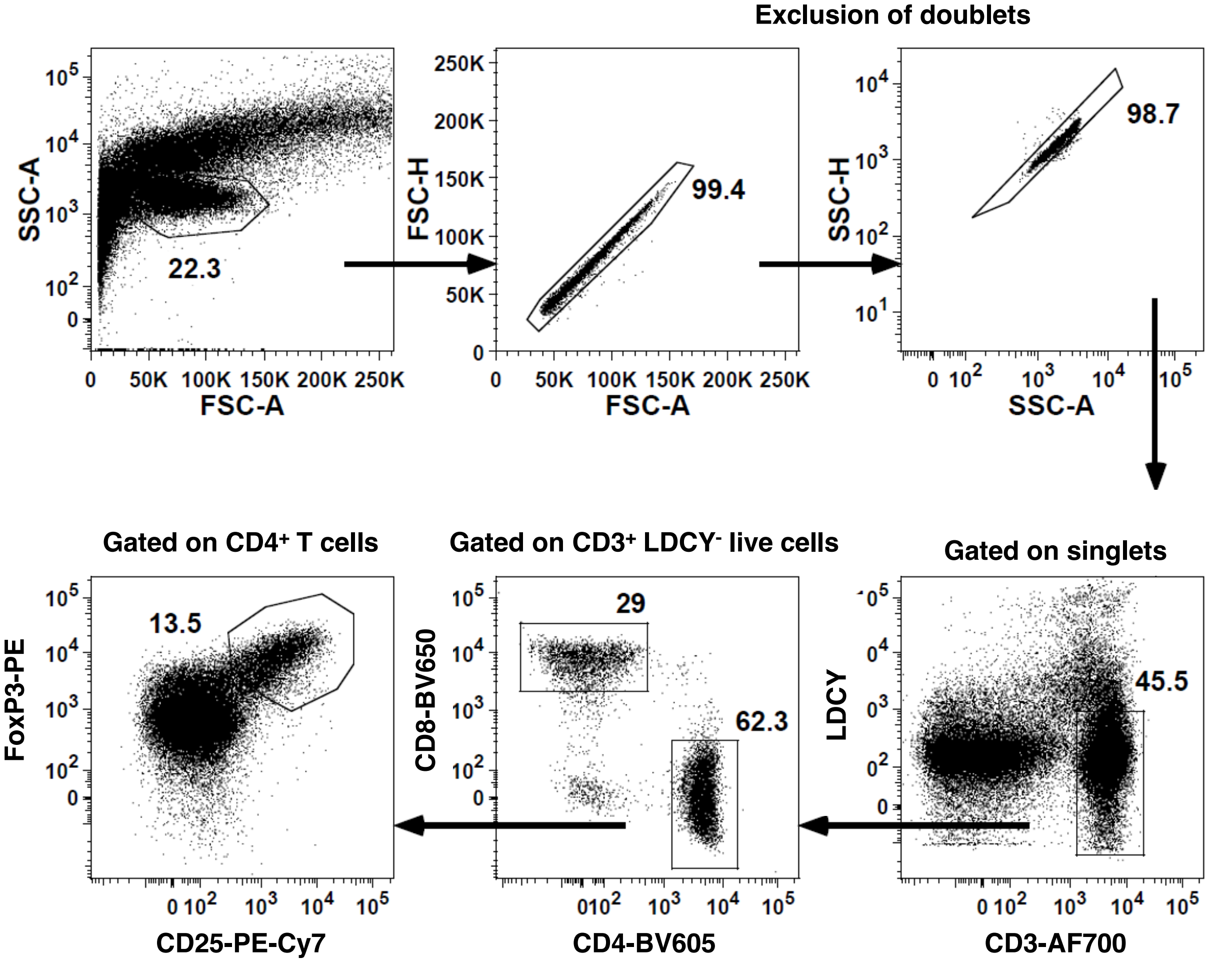

Supplementary Figure 1: Gating strategy for the phenotype characterization of T cell subsets by flow cytometry

Lymphocytes were defined according to their size (FSC) and granularity (SSC). Doublets (FSC-A *versus* FSC-H, and SSC-A *versus* SSC-H) and dead cells were excluded while gating on $CD3^+$ T cells, using Live Dead Cell marker (LDCY). The results are representative of one out of 34 tumors. Percentages of positive cells are represented on the dotplots. Abbreviations: FSC, Forward scatter; LDCY, Live dead cell yellow; SSC, Side scatter.

Supplementary Figure 2

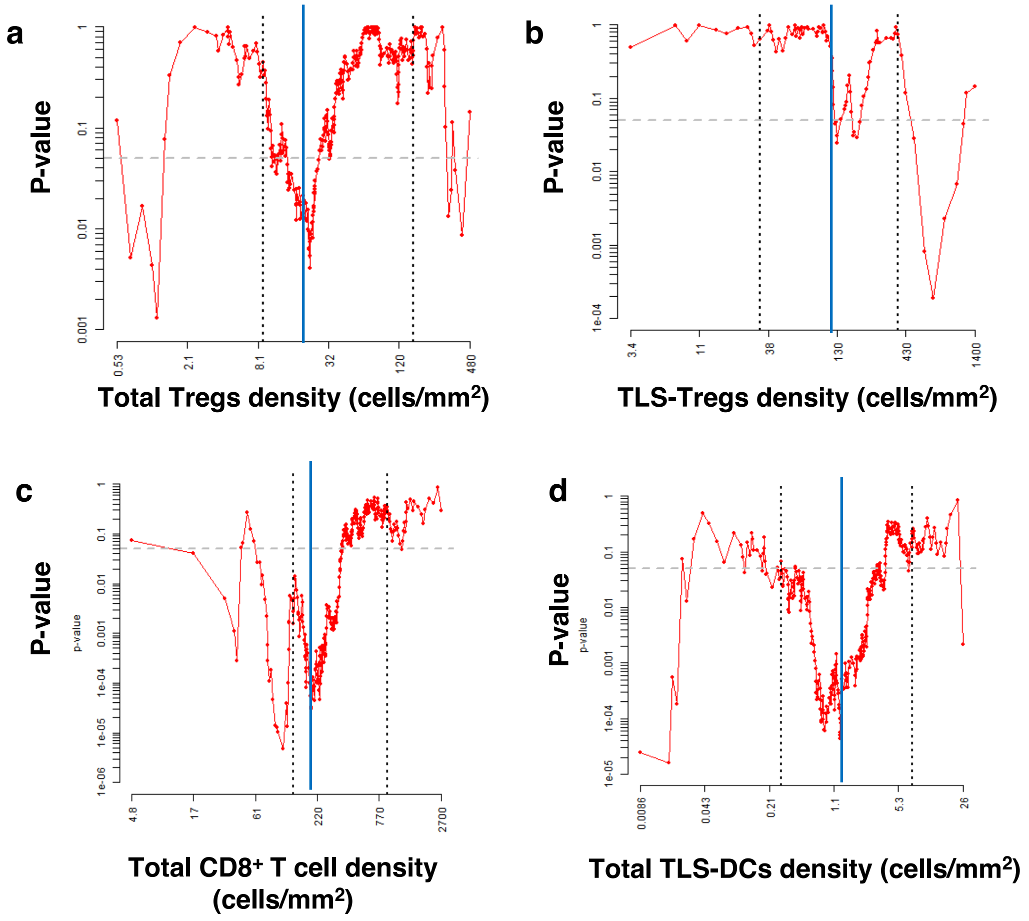

Supplementary Figure 2: Determination of optimal cutoff values for the discrimination of the high and low groups based on densities of the immune cells

The retrospective cohort of 338 NSCLC patients were stratified in two groups (high and low) based on the densities of total Tregs **(a)**, TLS-Tregs **(b)**, CD8$^+$ T cells **(c)**, and TLS-DC **(d)** using the optimal cut-off values. The optimal cut-off values (vertical blue line) are 21.93277 for Tregs, 127.0348 for TLS-Tregs, 191.177 for CD8$^+$ T cells, and 1.248 for TLS-DC. The X-axis from each graph shows the densities and the Y-axis shows the P-values. The horizontal dotted line shows the limit of P-value=0.05. The two vertical dotted lines show the values considered for the calculation of the optimal cut-off value with lowest P value. The densities falling outside of these two dotted lines are excluded during calculations.

Supplementary Figure 3

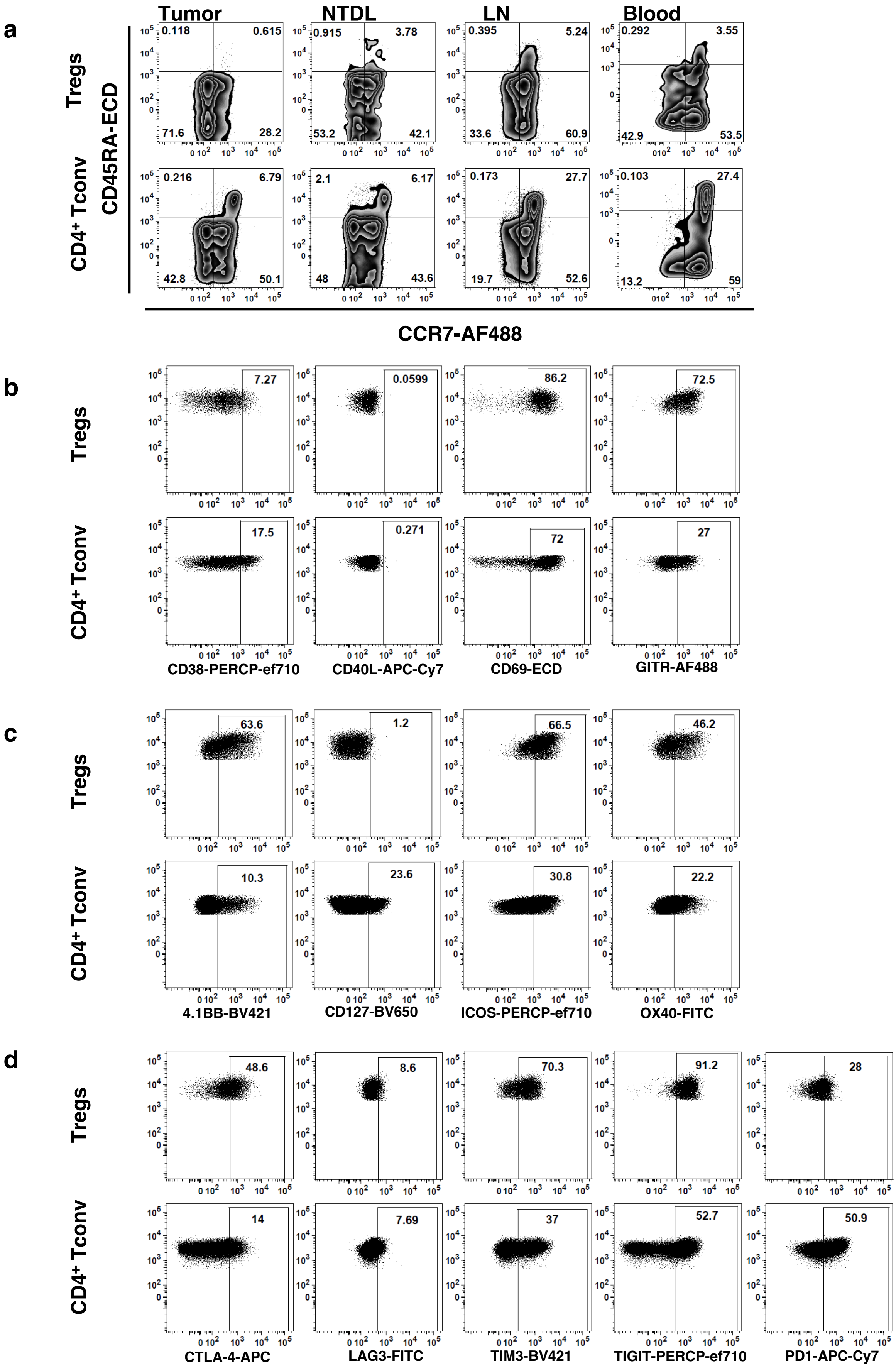

Supplementary Figure 3: Discrimination of the main four stages of differentiation of Tregs and $CD4^+$ Tconv in tumor and non-tumoral sites of NSCLC patients and expression of activation and immunosuppressive molecules

Analysis of the naïve (N, $CCR7^+CD45ra^+$), central-memory (CM, $CCR7^+CD45ra^-$), effector-memory (EM, $CCR7^-CD45ra^-$) and terminal effector-memory (TEMRA, $CCR7^-CD45ra^+$) subsets by Tregs and $CD4^+$ Tconv isolated from fresh tumor, non-tumoral distant lung (NTDL), lymph node (LN), and blood, based on the differential expression of CCR7 and CD45ra by flow cytometry (gated $CD3^+$ $CD8^-$) **(a)**. The results are representative of one out of 21 patients. Percentages of positive cells are represented on the dotplots.

The dotplots show the expression of activation (**b** and **c**) and immunosuppressive (**d**) markers by flow cytometry. The percentage of positive cells (black gated squares) was determined based on the isotype control. The results are representative of one out of 18 patients. Percentages of positive cells are represented on the dotplots among the total population of interest i.e. Tregs or $CD4^+$ Tconv cells.

Supplementary Figure 4

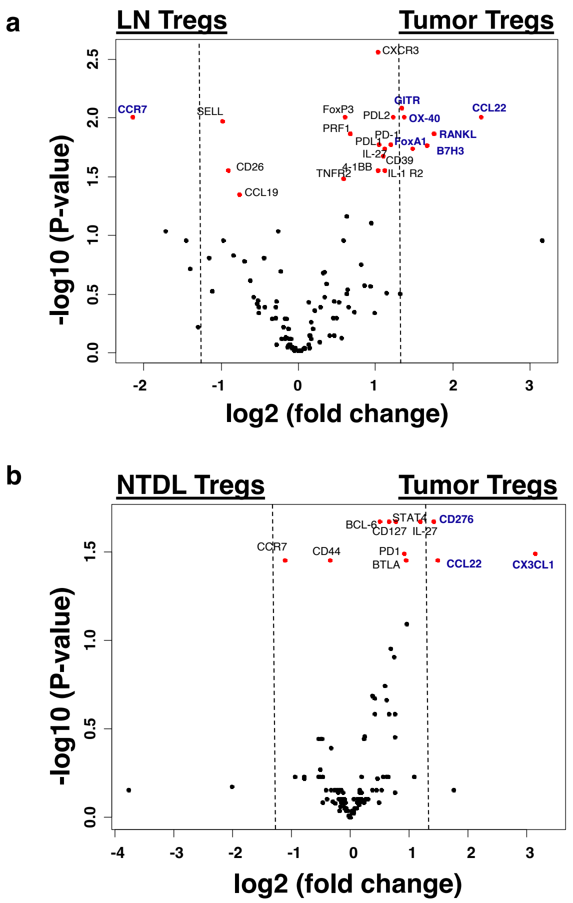

Supplementary Figure 4: Gene expression by Tregs from tumor *versus* non-tumoral sites

The volcano plots show genes that are over-expressed in Tregs isolated from tumor *versus* LN (panel **a**) and tumor *versus* NTDL (panel **b**) of NSCLC patients (n=20). The X-axis shows the log2 fold change values for each gene, and the Y-axis shows the –log10 (P-values). The P-value<0.05 (Student's t test) is considered significant, and genes showing a significant differential expression are highlighted in red dots. The red dots showing log2 fold change values higher than 1.2 are considered as significantly differentially expressed genes and are highlighted by their gene name in blue color.

Supplementary Figure 5

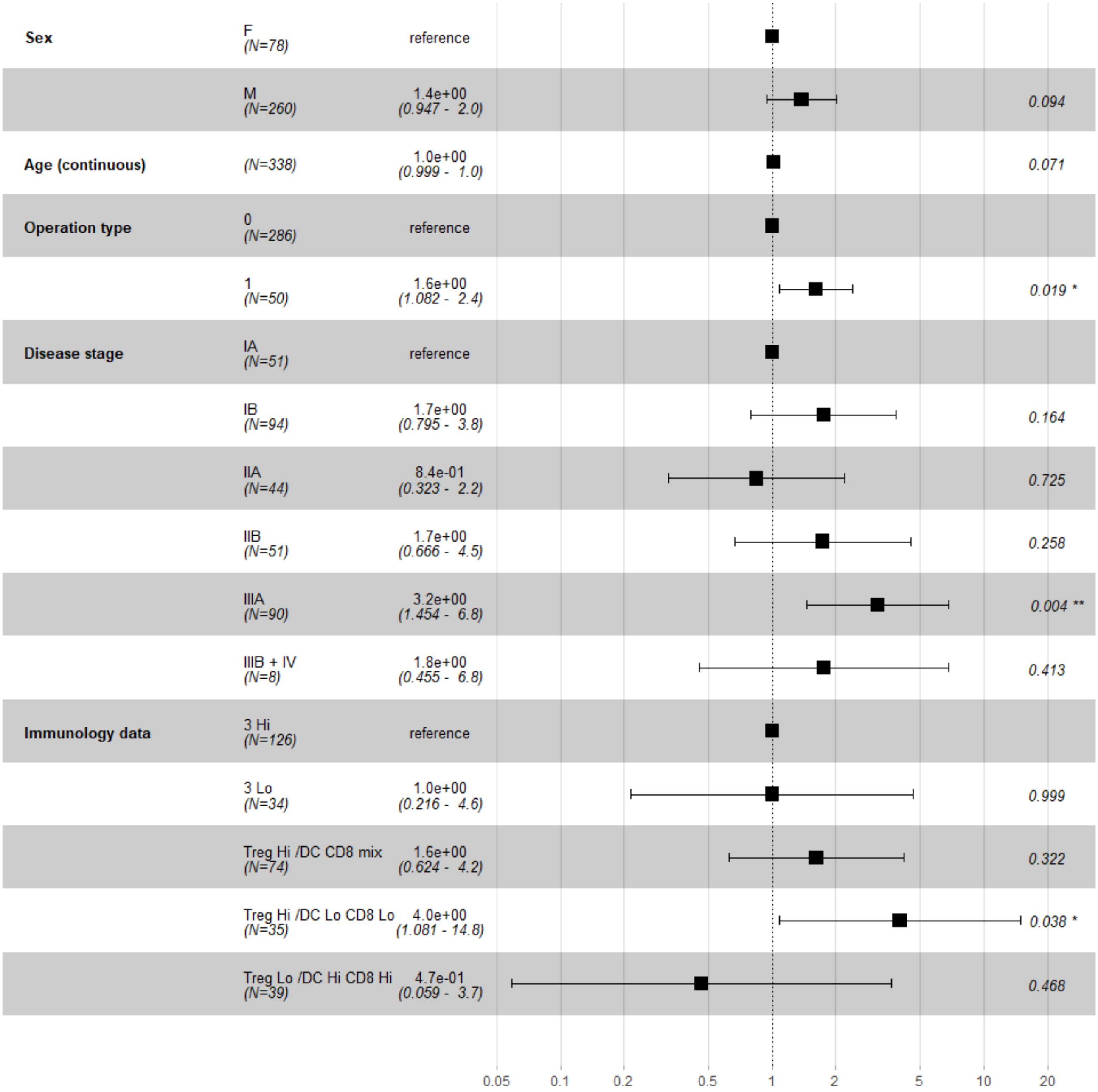

Supplementary Figure 5: Forest plot of adjusted hazard ratios (in line with Table 1)

Hazard ratios and p-values from adjusted multivariate CPH model. Codes for sex were F for female and M for male; codes for operation type were 0 for lobectomy and 1 for pneumonectomy. Infinite values from $Treg^{Lo}$ TLS-DC/$CD8^{mix}$ and interaction between NSCLC disease stages and immunology data were not displayed.

Supplementary Table 1

	Retrospective cohort		Prospective cohort	
**Characteristics**	No.	%	No.	%
**Gender**				
Male/female	260/78	77/23	38/22	63/37
**Age**				
Mean (years) +/- SEM	63.0+/-0.6		67.3+/-1.2	
Range	19-83		48-91	
**Smoking history**				
Current	274	81	40	67
Never smoker	46	14	11	18
ND	18	5	9	15
Pack-years (years)+/-SEM	41.9+/-1.4		37.8+/-4.5	
Range	0-120		0-140	
**Histological type**				
ADC	201	59	35	58
SCC	110	33	14	24
Others	22	7	8	13
ND	5	1	3	5
**Emboli**				
No	119	35	ND	ND
Yes	219	65	ND	ND
**pT stage**				
T1	67	20	7	11
T2	163	48	39	65
T3	83	25	12	20
T4	25	7	1	2
ND	0	0	1	2
**pN stage**				
N0	217	64	38	63
N1	61	18	10	17
N2	59	18	9	15
ND	1	0	3	5
**pTNM stage**				
I	145	43	29	48
II	95	28	8	14
III	96	28	20	33
IV	2	1	0	0
ND	0	0	3	5
**Vital status of patients**				
Alive	129	38	NA	NA
Dead	209	62	NA	NA

Supplementary Table 1: Clinical and pathological characteristics of the retrospective and prospective cohorts of NSCLC patients

All parameters were evaluated among 338 NSCLC patients of retrospective cohort and 60 NSCLC patients of prospective cohort. Pathologic staging of lung cancer was determined according to the new TNM staging classification.[52] Histological subtypes were determined according to the classification of the WHO.[53]

Abbreviations: ADC, adenocarcinoma; NA, not applicable; ND, not determined; SCC, squamous cell carcinoma.

Supplementary Table 2

Primary antibody	Host	Isotype	Clone	Dilution or working concentration	Company
CD3	Rabbit	IgG polyclonal	NA	1/80	Agilent
CD8	Rabbit	IgG polyclonal	NA	1/100	Spring biosciences
CD62L	Rabbit	IgG polyclonal	NA	5 μg/mL	Boster biological technology
Cytokeratins	Mouse	IgG1	AE1/AE3	1/50	Agilent
DC-Lamp	Rat	IgG2b	1010E1.01	1/250	Dendritics
FoxP3	Mouse	IgG1	236A/E7	1/80	Abcam
Secondary antibody					
anti mouse-uncoupled	Sheep	IgG1	NA	1/25	The binding site
anti-mouse IgG-AP	Goat	IgG polyclonal	NA	1/300	Jackson immuno-research
anti-rabbit – biotin	Donkey	IgG polyclonal	NA	1/200	Jackson immuno-research
anti-rat- biotin	Donkey	IgG polyclonal	NA	1/500	Jackson immuno-research
Amplification kit					
APAAP	NA	NA	NA	1/30	Agilent
Streptavidin-HRP	NA	NA	NA	1/300	Agilent
Substrate					
AEC	NA	NA	NA	3 drops/5mL	Vector laboratories
SAP	NA	NA	NA	3 drops/5mL	Vector laboratories

Supplementary Table 2: Antibodies and reagents used for immunohistochemistry

Abbreviations: AEC, 3-Amino-9-ethylcarbazole substrate; AP, Alkaline phosphatase; APAAP, alkaline phosphatase anti-alkaline phosphatase; APS, alkaline phosphatase substrate; HRP, Horseradish peroxidase; NA, not applicable.

Supplementary Table 3

Antibodies	Conjugate	Host	Isotype	Clone	Working concentration (μl/1 million of cells)	Company
4-1BB	BV421	Mouse	IgG1k	4B4-1	2	BioLegend
CCR7	AF488	Mouse	IgG2ak	G043H7	2	BioLegend
CD2	APC	Mouse	IgG2ak	S5.2	5	BD Biosciences
CD3	AF700	Mouse	IgG1k	UCHT1	1.5	BD Biosciences
CD4	BV605	Mouse	IgG2bk	OKT4	2	BioLegend
CD8	BV650	Mouse	IgG1k	RPA-T8	1	BioLegend
CD8	PerCP.Cy5.5	Mouse	IgG1k	RPA-T8	2.5	BioLegend
CD25	PE-cy7	Mouse	IgG1k	BD +	2	BD Biosciences
CD27	APC-A750	Mouse	IgG2a	CLB-27/1	1.5	Invitrogen
CD28	PerCPcy5.5	Mouse	IgG1	CD28.2	2	BD Biosciences
CD38	PerCP-eF710	Mouse	IgG1k	HB7	1	eBioscience
CD40L	APC-cy7	Mouse	IgG1k	24-31	20	BioLegend
CD45RA	ECD	Mouse	IgG1	2H4	1.5	Beckman Coulter
CD62L	APC	Mouse	IgG1k	DREG-56	2	BD Biosciences
CD69	ECD	Mouse	IgG2b	TP1.55.3	3	Beckman Coulter
CD127	BV650	Mouse	IgG1k	A019D5	3	BioLegend
CTLA-4	APC	Mouse	IgG1k	L3D10	3	BioLegend
FoxP3	PE	Rat	IgG2ak	PCH101	4	eBioscience
GITR	AF488	Mouse	IgG1k	AITR	2	eBioscience
ICOS	PerCP-eF710	Mouse	IgG1k	ISA-3	5	eBioscience
LAG-3	FITC	Mouse	IgG1k	17B4	10	EnzoLifesciences
OX-40	FITC	Mouse	IgG1k	ACT35	5	BD Biosciences
PD-1	APC-cy7	Mouse	IgG1k	EH12.2H7	4	BioLegend
TIGIT	PerCP- eF710	Mouse	IgG1k	MBSA43	2	eBioscience
TIM-3	BV421	Mouse	IgG1k	F38-2E2	3	BioLegend

Supplementary Table 3: Antibodies and reagents used for flow cytometry

Abbreviations: NA, not applicable.
