# Supplementary material for: Regulatory T cells infiltrate the tumor-induced tertiary lymphoïd structures and are associated with poor clinical outcome in NSCLC
Source: Commun Biol. 2022 Dec 24;5:1416. doi: 10.1038/s42003-022-04356-y (PMC9789959; doi:10.1038/s42003-022-04356-y)
Supplement: Supplementary file 3 — Description of Additional Supplementary Files [file 42003_2022_4356_MOESM3_ESM.pdf]

Description of Additional Supplementary Files

File name: Supplementary Data 1
Description: List of genes and accession number

File name: Supplementary Data 2
Description: Gene expression on T cell subsets (Figures 4 and 5a-c)

File name: Supplementary Data 3
Description: Clinical and immunological parameters of the retrospective cohort of NSCLC patients (Figures 8 and 9)

File name: Supplementary Data 4
Description: Phenotype of T cell subsets (Figure 2a)

File name: Supplementary Data 5
Description: Percentage of Tregs/total CD4+ T cells according to their anatomical sites (Figure 3a)

File name: Supplementary Data 6
Description: Stage of differentiation of T cell subsets according to their anatomical sites (Figure 3b)

File name: Supplementary Data 7
Description: Stage of differentiation of tumor-infiltrating Treg and conventional CD4+ T cells in TLS versus ouside TLS (Figure 3c)

File name: Supplementary Data 8
Description: Expression of ICP by tumor-infiltrating Treg and CD4+ conventional T cells (Figure 5d)

File name: Supplementary Data 9
Description: IFN-g and IL2 secretion secreted by CD4+ Tconv cultured with anti-CD3/anti-CD28 coated beads with or without autologous TIL-Tregs (Figure 6b)
